# Nanosecond T-Jump Experiment in Poly(glutamic acid): A Circular Dichroism Study

**DOI:** 10.3390/ijms13022239

**Published:** 2012-02-17

**Authors:** Lucille Mendonça, François Hache

**Affiliations:** Laboratory for Optics and Biosciences, Ecole Polytechnique/CNRS/INSERM, 91128 Palaiseau cedex, France

**Keywords:** circular dichroism, poly(glutamic acid), T-jump

## Abstract

Poly(glutamic acid) has been studied with a nanosecond T-jump experiment. A new experimental set-up based on the frequency-quadrupling of an 82 MHz Titanium-Sapphire laser allows rapid CD measurements to be performed. Combining time-resolved absorption and circular dichroism at 204 and 220 nm, we are able to measure precisely the unfolding relaxation time as well as the helical fraction evolution. We show that only CD at 220 nm is relevant to observe the unfolding of an alpha helix whereas no change is observed for CD at 204 nm. Conversely, both absorptions yield information on the dynamics of the process.

## 1. Introduction

Protein folding remains one of the most important challenges in modern biophysics [1]. The mechanisms by which an unfolded protein finds its way to its unique native conformation are still largely under debate. Even though the overall phenomena are now quite well understood, in particular with the existence of some funnel energy surface which guides the protein towards its final state [2], the microscopic details are not so well-known. In the last decades, it has become clear that formation of secondary structures (alpha helices, beta strands) often plays a determinant role as a first step in this folding pathway, creating some kinds of nucleation centers from which the folding propagates. It is therefore important to study the fundamental processes involved in the formation of such secondary structures.

Alpha helices are especially known to be the most rapidly-forming motif in proteins [3] and much work has been devoted to the study of the formation of such alpha helices [4]. In particular, the dynamics of this formation is of paramount importance because it directly results from the microscopic forces at stake in the protein. Experimental attempts to access this information have been developed in several groups. In most cases, the studies are conducted in *de novo* polypeptides which are simple enough to give reliable information [5,6]. Among these experiments, temperature-jump experiments have brought invaluable results [7]. By instantaneously increasing the temperature of the water surrounding the polypeptide and by monitoring the resultant denaturation process, these studies have evidenced ultrarapid dynamics in such systems. The main issue in these experiments is to come up with a reliable technique to investigate the folding state and more precisely the alpha helix content. The main technique utilized so far has been the absorption spectroscopy in the Amide I’ bands which lies in the mid-infrared. Indeed, the precise position of the Amide I’ bands is slightly dependent on the secondary structure. This technique has already brought many impressive results [8,9]. However, it suffers from a lack of quantitative estimation of the helix content. Because the IR bands are rather broad, the deconvolution of the absorption band is rather imprecise. A related technique is the UV resonance Raman technique which has been shown to yield quantitative results with a sophisticated Bayesian approach [10]. On the other hand, circular dichroism (CD) in the far UV has been known for a long time to give precise information on the secondary structures and CD spectra in this frequency domain are currently used to assess the content in secondary structures in unknown proteins [11]. This field of activity has been even more active for a few years with the development of the SRCD measured with synchrotron radiations [12]. In particular, alpha helices display a characteristic CD spectrum with two minima at 222 and 208 nm. In the case of simple peptides involving only alpha helices or random coil, measuring the CD at 222 nm is known to be very quantitative and Rohl *et al.* [13] have established the following relation expressing the helical fraction *f_H_* in the peptide as a function of the measured molar ellipticity [*θ*]_222_ per residue:

(1) $$f_{H}=\frac{{\left[\theta \right]}_{222}-{\left[\theta \right]}_{C}}{{\left[\theta \right]}_{H}-{\left[\theta \right]}_{C}}$$

where [*θ*]*_C_* = 2220 − 53*T* is the random coil ellipticity for temperature T expressed in °C and [*θ*]*T_H_* = − 44000 + 250 is the ellipticity of an infinite alpha helix. Ellipticities are expressed in deg·cm^2^·dmol^−1^ per residue. From this above expression, it is clear that monitoring the dynamics of the CD following a T-jump excitation allows one to gain precise quantitative information on the evolution of the sample’s helicity.

We have developed such an experiment capable of monitoring the dynamics of the CD in the far UV in a T-jump experiment. CD measurement is achieved by directly making the difference in absorption for a left and a right-circularly polarized light, according to its definition. This technique is in fact the basic one utilized in commercial systems, except that here the time-resolved character prevents the use of a rapid modulation technique as usually done. We have carried out this experiment on a poly(glutamic acid) (PGA) which is known to fold in alpha helices with an helical content strongly dependent on the temperature [14]. Similar peptides have already been studied with IR probes with the above-mentioned limitations [15]. With our technique, we bring quantitative information to this problem. We have monitored the variation of CD and absorption at 220 and 204 nm in the hundreds of nanoseconds after the T-jump and we show that both results agree and allow us to observe an ultrafast denaturation of the PGA with a time constant of 1.2 μs. Interestingly, we verify that information about helicity is clearly given by the ellipticity at 220 nm whereas no such information is obtained at 204 nm where helices and random coils display the same CD values.

## 2. Results and Discussion

### 2.1. Steady-State Spectra

**Figure 1:**
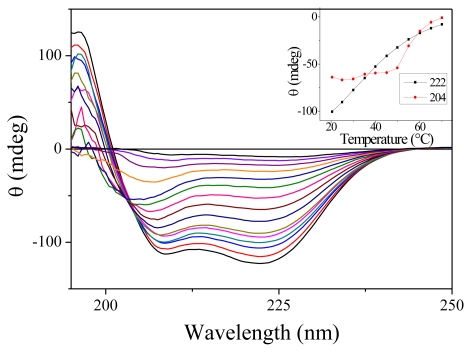
Circular Dichroism (CD) spectra of Poly(glutamic acid) (PGA) for 15 temperatures ranging from 7.7 to 70 °C. For this measurement, the PGA concentration was reduced to 7.5 mg/mL and the cell thickness was 100 μm. The inset shows the ellipticity measured at 204 and 222 nm as a function of the temperature.

We have carried out measurement of the steady-state absorption and CD spectra of the PGA samples as a function of the temperature. As expected for this non-aromatic peptide, the onset of absorption occurs around 230 nm. Only slight changes in absorption are observable as a function of temperature [16]. CD spectra are much more temperature-sensitive. Figure 1 shows the CD spectra for 15 temperatures between 7.7 and 70 °C. At low temperatures, the spectra display the characteristics double dip structure assignable to the alpha helix secondary structure. When temperature increases, the fraction of helices decreases as expected. Note that there is no abrupt change denoting a phase transition as observed for proteins but only a smooth decrease. This feature is well established for homopeptides such as polyalanine [17]. The inset in Figure 1 shows the variation of CD with temperature for the two wavelengths that we have investigated. Around 20 °C, we measure in terms of molar ellipticity Δ[*θ*_222_] = 420 Δ*T*.

An interesting feature of these CD spectra is the appearance of an isodichroic point around 204 nm for temperature below 50 °C. However, as can be seen from the slight slope in the inset of Figure 1, all the curves do not cross exactly at the same point. Furthermore, the CD spectra at high temperature are clearly different. These features probably indicate that the two-state model is not adapted to describe the dynamics of PGA. Such a conclusion has already been reached by others [18].

### 2.2. Experiments at 220 and 204 nm

T-jump experiments were carried out at two wavelengths following the techniques described in the experimental section. Actually, the raw data given by the experiment consist in the time-resolved transmission of the sample which we translate into a change in absorption. Access to CD is then performed by comparing the data for a left or a right circularly polarized beam. It is therefore obvious that data about absorption will be easier to obtain and consequently have a much better signal-to-noise ratio than CD ones.

**Figure 2:**
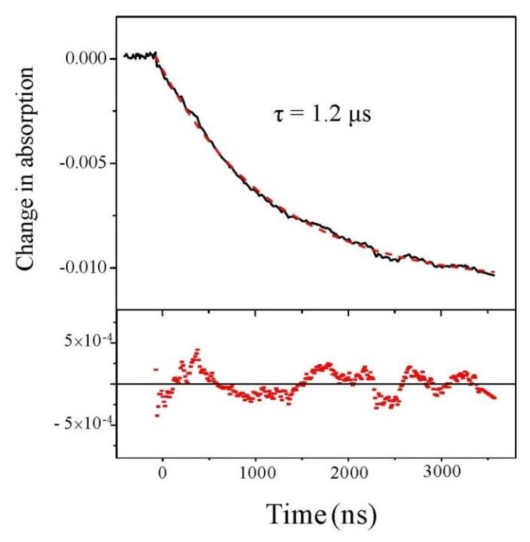
Change in absorption at 220 nm as a function of time. The dashed line is a monoexponential fit whose residual is displayed in the lower panel.

We first analyze the change of absorption at 220 nm, independently of the beam polarization. Results are displayed in Figure 2 for a starting temperature of 21 °C and an experimental T-jump of 4 °C. We observe a decrease in the absorption at this wavelength. This decrease in absorption is consistent with a temperature rise of 4 °C [19]. The nice feature about this signal is its excellent quality which allows us to carry out a fit and we see that fitting with a single exponential curve gives a very good result.

**Figure 3:**
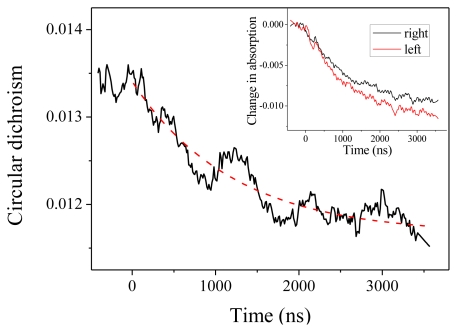
Circular dichroism in absolute value measured at 220 nm as a function of time. The inset shows the corresponding changes in absorption for the left and right circular polarization.

We come now to the CD measurements. In our experiments, what we measure is actually *(α_L_* *–α_R_)L*, where *α_L_* (*resp.α_R_*) is the absorption coefficient for a left (*resp.* right) circular polarization and *L* is the optical path. With this definition, CD at 220 nm is about −0.0135 at room temperature. As can be seen in Figure 3, we observe a decrease (in absolute value) of the CD down to about −0.0115. This CD change is obtained by comparing the relaxation curve for the absorption for a left or a right polarized probe beam, as depicted in the inset of Figure 3. Both curves display the same time constant within the measurement accuracy, but with different amplitudes. The dashed line in Figure 3 is the reproduction of the exponential fit obtained from the absorption measurement, which confirms that both effects are two signatures of the same unfolding process. The change in CD corresponds to a change in molar ellipticity of 1860 deg·cm^2^·dmol^−1^. Applying the above-derived expression Δ[*θ*]_222_ = 420 Δ*T*, this corresponds to a temperature increase by 4.4 °C, in agreement with the expected T-jump.

**Figure 4:**
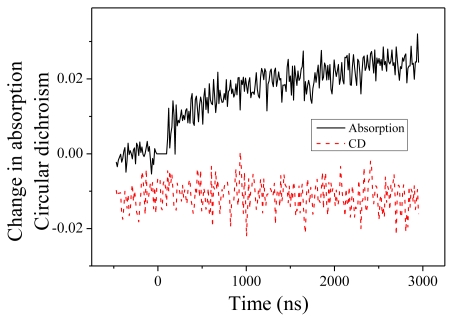
Change in absorption and circular dichroism measured at 204 nm as a function of time.

We present now the measurements performed at 204 nm. Change in absorption and CD are displayed in Figure 4. Two features are noteworthy. First, the change of absorption is in the opposite direction compared to 220 nm. At 204 nm, we observe an increase of the absorption. This feature is consistent with the measured absorption spectra as a function of the temperature. The time constant of this change, equal to 1.2 ± 0.2 μs, is similar to what we measured at 220 nm. This indicates that both absorption changes as well as CD changes originate in the same fundamental denaturation process. Second, we observe no CD dynamics at this wavelength. This is in perfect agreement with the steady-state CD spectra displayed in the Figure 1 where one observes an isodichroic point at 204 nm when the temperature changes from 20 to 50 °C.

### 2.4. Discussion

The most interesting feature of these results is the complementarity of the CD and absorption measurements. Thanks to CD measurements, we can ascertain that we observe an unfolding of the alpha helices in PGA following the 4 °C T-jump. This is made clear by the decrease (in absolute value) of the CD at 220 nm. This CD at 220 nm furthermore allows us to give a quantitative estimate of the helical fraction. Let us recall that the molar ellipticity per residue is connected to our measurement by

$$[\theta ]=\frac{3298}{ln10}\frac{CD}{cL}$$

where *c* is the molar concentration of residues (in *M*) and *L* the sample thickness (in cm). The “*ln10*” factor is necessary because as explained above we define the CD as a difference in absorption and not in absorbance. With the help of the formula recalled in the introduction, we calculate that the helicity of our PGA sample at room temperature is 34%. When the temperature shifts to 25 °C because of the T-jump, there is a slight change of the CD even if the helicity does not change, due to the temperature-dependence of [*θ*]*_H_* and [*θ*]*_C_*. However, this CD change is very small and we can assign the decrease in CD to the unfolding of the peptide. We calculate the final helicity to 30%. This conclusion is also supported by the invariance of the CD at 204 nm. If the observed changes were due to some thermal effect, one could expect that the CD at 204 nm would change also. However, if the change is due to a partial denaturation of PGA, one expects a change in CD at 220 nm but no change at 204 nm, where the temperature-dependent CD spectra displays an isodichroic point.

Beside this quantitative estimate of the peptide helicity, the main interest of this experiment is to determine the time constant of the denaturation process. Examining Figure 3, we indeed observe the relaxation of the CD, but due to the extreme smallness of such signal, the curve is very noisy. One should average a lot to strongly decrease this noise. However, in our case, it is not necessary because the absorption curves have a much better signal-to-noise ratio and we can extract the relevant dynamical information from those curves. This is particularly clear in Figure 2, which displays the change in absorption at 220 nm. This curve was averaged over 5 h to give a very good signal. We can see that the relaxation is very well fitted by a single exponential with a time constant equal to 1.22 ± 0.01 μs, which corresponds to the denaturation time at *T* = 25 °C. This relaxation time is of course very dependent on the temperature, but this value is in very good agreement with Reference [15] in which a relaxation time of 1.44 μs at 27 °C is measured for a comparable pH. Note that these authors also can fit their data with a single exponential decay.

Monitoring the CD in the far UV is therefore a very efficient way to investigate the unfolding dynamics in peptides or proteins. Because of the clear assignment of CD structures in this wavelength range, the time-resolved CD experiments allow the study of the dynamics of peptide’s secondary structures with a very good time resolution.

## 3. Experimental Section

### 3.1. Sample

The experiment has been carried out with a poly(glutamic acid) (PGA) sample. The peptide was purchased from Sigma-Aldrich and dissolved in water without further purification. The molar weight in this sample is 64,000, corresponding to about 500 residues per peptide. The concentration is 20 mg/mL, which corresponds to concentration of 0.155 M in terms of glutamic acid residues.

The pH needs to be carefully controlled. Indeed, depending on the pH, PGA can be completely folded into alpha-helices or display a random coil structure. Because we want to have a sample whose conformation changes with temperature, we chose a pH of 4.8, which was adjusted by careful addition of acetic acid and sodium acetate (Sigma-Aldrich). The final acetate concentration is about 0.3 M. At this pH, absorption as well as circular dichroism display a strong temperature dependence which is a signature of a change in the helical content of the sample.

### 3.2. Steady-State CD Measurements

Temperature-dependent CD spectra were measured with a commercial spectropolarimeter Jasco J710. For these measurements, the PGA sample was diluted down to 7.5 mg/mL. The pH was kept at 4.8. The measurements were carried out in a 100 μm thick cell. The cell temperature was controlled by a Peltier regulator.

### 3.3. T-Jump Measurements

For the T-jump measurements, the peptide is placed into a 100 μm path length quartz cuvette (Hellma). This cell can be thermalized by external water circulation. Two experimental components are necessary to carry out this experiment: one needs a nanosecond IR source to intitiate the temperature jump and a far-UV system capable of measuring the CD with a good time resolution.

Initiation of the T-jump in the sample is usually made with a nanosecond Nd:YAG laser. It can be achieved through indirect or direct heating. Indirect heating consists in adding into the sample a dye which absorbs the second harmonic of the Nd:YAG laser (typically Basic Red). Although efficient and cheap, this solution is however prone to many cavitation problems. Indeed, the dye molecules act as nucleation centers for the growth of vapor bubbles. This cavitation issue is much less pronounced with a direct heating which consists in directly heating the water and which results in a homogeneous heating. In our experiment, the solvent is neat water, contrarily to much more common experiment dealing with an IR probe which necessitates to use heavy water. Direct heating of water can be achieved through the excitation of the ν_1_ + ν_3_ overtone mode which takes place at 1.5 μm. The most popular means to attain this wavelength are the Raman shifter and the dye laser. We have chosen another technique and implemented a nanosecond OPO pumped by the second harmonic of the Nd:YAG laser [19]. This device allows us to finely tune the pump wavelength to optimize the water absorption. Maximum water heating was achieved for λ = 1.45 μm, with an absorption equal to α = 32 cm^−1^. The pump energy is currently about 7 μJ. This IR beam is focused down to a waist of about 500 μm. With these numerical parameters, we can estimate from the energy absorbed by the water a temperature increase by 5 °C in a 100 μm thick cell. Indeed, we have been able to measure the T-jump in our set-up by means of a Bromothymol Blue/Tris-HCl mixture and we have obtained T-jump of 4 to 6 °C. The dynamics of this T-jump has also been fully characterized: the temperature remains constant for up to 700 μs and rotation of the cell at 300 rpm insures that each pump pulse (repetition rate = 30 Hz) excites a fresh volume in the sample, without any cumulative heating.

**Figure 5:**
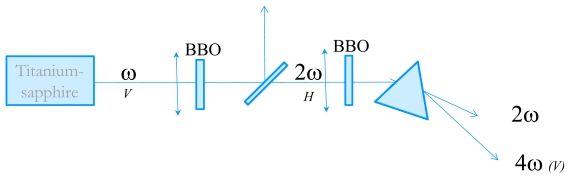
Schematic of the fourth harmonic generation of the 82 MHz Titanium-Sapphire laser. The letters *V* or *H* refer to the beam polarizations.

In order to measure the time-resolved circular dichroism in the UV, we have developed an experimental technique very close from the one proposed by Wen *et al.* [20]. The starting point is an 82 MHz Titanium-Sapphire laser delivering 3–6 nJ, 100 fs pulses with wavelengths tunable between 800 and 900 nm. We then perform fourth-harmonic generation to obtain wavelength in the far-UV. The tunability in the UV is from 202 nm (limited by phase-matching issues) to 225 nm. The experimental set-up is described in Figure 5. Second harmonic generation is performed in a 1 mm thick Type I BBO crystal cut at 30°. The pulse energy is about 0.3 nJ. The choice of the BBO thickness is dictated by the trade-off between conversion efficiency and control of the 2ω pulse duration. Indeed, choosing a thicker crystal results in a higher energy but also to a much longer pulse due to group-velocity dispersion which is not favorable for the fourth harmonic process. The 4ω pulses are obtained by frequency-doubling of the 2ω ones in a 2 mm thick BBO crystal cut at 60°. Because of phase-matching issues, the two BBO crystals are oriented perpendicular to each other. The beam polarizations are depicted in Figure 5. The 4ω pulses are then spatially separated from the 2ω ones by a fused silica prism. The 4ω beam is then sent to the CD experiment. The pulse energy is very low but its fluorescence on a paper is visible with the naked eyes and it can be easily detected with a photomultiplier tube (PMT).

The 82 MHz UV beam is sent through the sample and detected by a rapid PMT whose output is monitored by a 500 MHz oscilloscope. The oscilloscope is directly triggered by an electronic signal synchronized with the pump pulses so that all the oscilloscope traces display the same time interval relative to the pump onset. We can therefore average the oscilloscope traces and directly obtain the signal dynamics from the oscilloscope on a timescale adjustable in the micro or millisecond range. In order to measure the CD, the UV polarization is alternately fixed to left or right circular with a Pockels cell onto which we apply ±500 V. The traces are then stored and processed with a personal computer. Averaging of the left and right traces gives the variation of the absorption as a function of the time elapsed after the excitation whereas the difference between the two traces provides the dynamics of the CD. In our experiment, CD is directly achieved by making the difference of the transmitted signals for the two circular polarizations. In commercial apparatus, this difference is carried out through the modulation of the polarization with a photoelastic modulator and a subsequent demodulation of the transmitted signal. However, this is not possible here because we want to access the dynamics of the signal, which is not compatible with the integration performed in demodulation devices such as lock-in amplifiers.

Carrying out the experiment in the far UV brings two differences with usual T-jump experiment coupled with an IR probe. First, we can use regular water instead of heavy water. Beside the practical point of view, using natural water allows the study of proteins close to their physiological conditions. Second, we use fused silica cells which have a good transparency in the UV, instead of CaF_2_ ones for IR transmission. Because silica cell have a less good heat conductivity, it avoids a too rapid cooling of the water and it allows the temperature rise to be conserved for a longer time, preventing interference between the protein dynamics and the cooling process.

## 4. Conclusions

We have presented experimental results on the thermal denaturation of PGA. By employing a 4 °C T-jump, we have evidenced the unfolding of the alpha helices. Thanks to CD measurement at 220 nm, we have been able to quantify the unfolding and to show that the helicity drops from 34 to 30% when the temperature goes from 21 to 25 °C. By using the complementarity of the absorption and CD curves, we have also been able to measure precisely the time constant of this denaturation process to 1.22 ± 0.01 μs. We have also verified that no CD change is observable at 204 nm where folded and unfolded peptides have the same CD signals, discarding any artifact in the measurement.

Thanks to the simplicity of this set-up, monitoring the content in alpha helices of peptides and proteins with a time resolution down to 10 ns will become easy to perform and should bring invaluable information on the elementary processes of folding.
